# Effect of Number of Players and Maturity on Ball-Drills Training Load in Youth Basketball

**DOI:** 10.3390/sports5010003

**Published:** 2017-01-02

**Authors:** Daniele Conte, Terence Favero, Meike Niederhausen, Laura Capranica, Antonio Tessitore

**Affiliations:** 1 Department of Movement, Human and Health Science, Univesity of Rome “Foro Italico”, 00135 Rome, Italy; laura.capranica@uniroma4.it (L.C.); antonio.tessitore@uniroma4.it (A.T.); 2 Department of Biology, University of Portland, Portland, OR 97203, USA; favero@up.edu; 3 OHSU-PSU School of Public Health, Oregon Health & Science University, Portland, OR 97239, USA; niederha@ohsu.edu

**Keywords:** small-sided games, basketball conditioning, basketball training, maturation status

## Abstract

This study aimed to assess the basketball ball-drills workload analyzing: (1) the effect of varying the number of players involved on physiological and technical demands; (2) the temporal changes in players’ responses across bouts; and (3) the relationship of players’ workload with their maturation status and training age. Twelve young male basketball players (mean ± SD; age 13.9 ± 0.7 years; height 1.76 ± 0.06 m; body mass 65.7 ± 12.5 kg; HR_max_ 202 ± 8 beat·min^−1^) completed three bouts of 4 min interspersed by 2 min of passive recovery of two vs. two and four vs. four ball-drills. The mean percentage of HR_max_ (%HR_max_) and ratings of perceived exertion (RPE) were collected. Technical actions (TAs) (dribbles, passes, shots, interceptions, steals, rebounds, and turnovers) were calculated through notational analysis. Players’ genitalia development (GD) and pubic hair (PH) growth were assessed using Tanner scale. Results showed a higher %HR_max_ (*p* = 0.018), RPE (*p* = 0.042), dribbles (*p* = 0.007), shots (*p* = 0.003), and rebounds (*p* = 0.006) in two vs. two compared to four vs. four condition. Furthermore, a statistical difference was found for %HR_max_ (*p* = 0.005) and number of passes (*p* = 0.020) between bouts. In addition, no correlation between GD, PH, and training age with %HR_max_, RPE, and TAs was found. These findings suggest that variations of the number of players involved affect ball-drills workload and that ball-drills training intensity varies across bouts. Finally, ball-drills elicit an adequate training stimulus, regardless of players’ maturation status and training age.

## 1. Introduction

Game-based drills are training methodologies often used by team sports coaches. Such sport-specific conditioning has been found to develop players’ physical and physiological demands and both technical and decision making skills [1]. Ball-drills (BD) conducted as part of small-sided games have been widely investigated in several team sports such as soccer [2,3,4,5], rugby [6,7], handball [8,9], futsal [10], and basketball [11,12,13,14,15]. In particular, studies on basketball showed a comparable response in the physiological demand (above 85% of HR_max_) between game-based training and official games [11], underlining that this methodology offers a sport-specific training stimulus. The physiological and technical demands in basketball BDs have been shown to vary significantly when using different numbers of players [11,13,14], court sizes [14], game rules [12], and work-to-rest ratios [14].

The number of players involved has been reported to be the main factor influencing the physiological and technical demands in basketball BDs [11,13,14]. A reduction of the number of players involved while maintaining the same playing area corresponded to a moderately higher physiological demand in basketball BDs [11,13,14]. Moreover, a moderate to strong increase of the technical demand (i.e., dribble, pass, shot, rebound, ball screen) in BDs played with two vs. two compared to four vs. four players has been demonstrated [13,14].

The analysis of workload changes across bouts of the same BD could also provide additional interesting information to help coaches in designing sound training programs. Changes in physiological and technical demands across bouts have been extensively studied in soccer small-sided games (SSGs) [16,17,18], while only one study investigated the changes of training loads across bouts in basketball BDs [11]. This research documented no statistically significant differences in the physiological demands between three 4-min bouts interspersed by 3 min of passive recovery. However, this study provided information only on the physiological demands, while to the best of our knowledge, no previous study analyzed the changes in technical demands across bouts in basketball BDs.

Considering that studies in basketball BDs mainly investigated young players in a chronological age after the completion of their maturation process (i.e., >15 years old) [11,13,14], a multifactorial approach to assessing the physiological and technical demand of basketball BDs in younger players would be important for the body of sport literature. Previous studies showed that growth and maturation are fundamental concepts to better understand the identification, selection, and development processes of young athletes [19,20]. In fact, the maturation status characterizes young athletes’ physical and anthropometric features, and it is an important consideration in developing effective age-based training programs [21]. Usually, young players are above the mean for their age in height and mass, and tend to be advanced in biological maturity in elite development programs [22]. Previous investigations documented that team sport players’ maturity status affects the absolute maximal strength outputs as a consequence of the growth in body size and muscle mass [21]. Furthermore, maturation status has been shown to affect body size and upper body strength (grip strength and medicine ball throw) in 14–15-year-old basketball players [23]. Moreover, skeletal maturity has been documented as one of the main predictors for maximal short-term power output measurement such as line drill test, Bangsbo sprint test, and Wingate anaerobic test [24]

Variation in sexual maturity was also investigated in relation to basketball technical skills [23]. This study demonstrated that maturation status significantly influenced two time-based technical skills tests (dribbling and defensive movements) in 14-year-old basketball players. However, in that study, technical skills were performed individually without the interaction with other players, rather than in game-based conditions like BDs. Regarding collective drills, da Silva et al. [16] examined the relationship between maturation status and physiological and technical parameters in young soccer players performing different settings of small-sided games, showing no association between them. However, to our knowledge, no studies have investigated the effect of maturation status in basketball BDs. Considering that youth basketball teams are usually composed of players with different maturation levels, it is important to assess whether differences in players’ maturity status may influence physiological and technical demands during basketball BDs.

In many youth basketball teams, players with different levels of experience coexist. This difference could be responsible for different players’ physiological and technical demands. In fact, more experienced players might have a higher skill level and therefore be more involved in game situations than less experienced players. To the best of our knowledge, no previous investigations assessed the effect of different playing experience on BDs workload, calling for further studies in this area. Therefore, this study has multiple purposes, specifically to analyze: (1) the effect of varying the number of players involved on physiological and technical demands in basketball BDs in young players (i.e., <15 years of age); (2) the workload changes throughout bouts; (3) the relationship of BD physiological and technical demands with players’ self-assessed maturation status and training age.

Based on previous investigations analyzing the physiological and technical demands in basketball BDs [11,13], it has been hypothesized that a reduction of the number of players involved will increase training load, and that workload will be lower in the first bout compared to the subsequent ones. Furthermore, since maturation status might influence BDs physiological and technical demands, it has been hypothesized that players’ self-assessed maturation status and playing experience will negatively and positively correlate with the physiological and technical demands, respectively.

## 2. Materials and Methods

### 2.1. Participants

Twelve (eight perimeter and four post players) young male basketball players (mean ± SD; age 13.9 ± 0.7 years; height 1.76 ± 0.06 m; body mass 65.7 ± 12.5 kg; HR_max_ 202 ± 8 beat·min^−1^) volunteered to participate in this study. All players were members of an “Under 15” team participating at a regional-level of the Italian youth basketball championship. The study was conducted within two weeks during the 2013/2014 season. Players were familiarized with the procedures during the pre-season period, while the experimental protocol took place during the last macrocycle of the in-season period (March–May). Players completed a similar pre-season and in-season volume of training before the starting of the experimental procedures. Players performed three 90-min training sessions and one official game per week. Selection criteria for recruitment in the study included the absence of injury in the past 6 months. Ethics approval was obtained from the Institutional Review Board for Research involving Human Subjects of the University of Portland, approval number IRB00006544. Written informed consent was obtained from all the subjects and their parents after a detailed explanation of the aims of the study.

### 2.2. Procedures

The number of players involved and bouts performed served as the independent variables, while the heart rate (HR), ratings of perceived exertion (RPE), and technical actions (TA) were the dependent ones. Moreover, the relationship between the dependent variables and players’ maturation status and playing experience was evaluated in each BDs setting.

BDs were composed of three bouts of 4 min interspersed by 2 min of passive recovery, and were played during regular training sessions after 15 min of standardized warm-up. This set-up was selected because it represents one of the most used basketball practice drills with a duration (12 min) similar to that reported in previous studies [11,12,13], and with a sport-specific work-to-rest ratio [25,26]. Only physiological and technical demands during BDs live time were analyzed, while stoppage time was not considered. BDs were separated by at least 24 h and performed at the same time of the day (5 p.m.) to avoid any effect of circadian rhythms on the measured variables. All subjects played the two vs. two and four vs. four BDs in random order on three separate consecutive sessions. Players were not involved in more than one BD condition in the same training session and were allowed to hydrate ad libitum. BDs were played on full court, and only man-to-man defense system was allowed to standardize technical-tactical parameters. Moreover, to avoid possible interruptions during the play, extra balls were placed around the boundary lines to replace the ball as soon as out of the play. Each drill was refereed by the same component of the coaching staff, which was qualified to referee. In case of fouls, no free throws were awarded, and the game was restarted with a throw-in. For each BD, coaching staff formed balanced teams based on players’ skills and play positions, and provided continuous verbal encouragement to participants.

### 2.3. Measures

Before participating in the study, players’ anthropometric measurements (height and body mass) were collected. Moreover, all players completed the 30–15 Intermittent Fitness Test (30-15IFT) specifically developed for basketball [27] to assess their HR_max_ (beat·min^−1^). The test protocol involved 30-s shuttle runs interspersed by 15-s passive recovery periods executed on the full basketball court (28 m) at an increasing pace governed by a pre-recorded beep [27]. Players were asked to run back and forth on the basketball court and to complete as many stages as possible. The test ended when the players could no longer maintain the required running speed and the heart rate at that time was registered as HR_max_. Heart rate beats were recorded at 1-second intervals using HR monitors with internal memory (Team System 2, Polar, Kempele, Finland) and exported and analyzed using Excel software (Microsoft Corporation, Redmond, WA, USA). Ball-drill HR data were expressed as a percentage of HR_max_ (%HR_max_). Moreover, players’ rating of perceived exertion (RPE) was collected at the end of each BD using the modified CR10-scale [28] with which players were previously familiarized.

Technical actions (TA) were assessed using notational video analysis through the Kinovea software (www.Kinovea.org, accessed on 15 March 2015) This methodology has been previously used and described in previous investigations [12,13]. The frequency of occurrence of the following TAs was also collected: dribble, pass, shot, interception, steal, rebound, and turnover. The reliability of the video analysis was determined by having a second experienced analyst repeating it for each player for one of the studied drills. Thus, the coefficient of variation (CV) for the difference between repeated measurements was calculated, and results reported a good reliability with CV of 1%–4%.

Maturity was assessed by means of a self-administered Tanner scale [29], which has been shown to be a valid method to evaluate the maturity status of adolescent athletes [30]. Players received a sheet with standard illustration based on five stages for pubic hair (PH = from PH1 to PH5); and genitalia development (GD = from GD1 to GD5) based on Tanner sexual maturity evaluation [29]. Players were then required to move into an isolated and closed room and circle the drawing that best represented their PH and GD status. The number of players for each PH and GD statuses were: *n* = 4, 6, and 2 for PH3, PH4, and PH5, respectively; and *n* = 5, 4 and 3 for GD3, GD4, and GD5, respectively. In addition, participants’ playing experience was asked. It was defined as the period in which players were involved in structured basketball training sessions.

### 2.4. Statistical Analysis

Mean and standard deviation (SD) were calculated for all the dependent variables. Normality and homogeneity of variances were assessed using the Shapiro–Wilk test, showing a normal distribution for physiological parameters, while RPE and TAs data were not normally distributed. Therefore, a two × three (number of players × bouts) two-way ANOVA for repeated measures was applied to assess differences between two vs. two and four vs. four and the three bouts for %HR_max_. When significant F values were found for the bout factor, Bonferroni post-hoc tests were used. In addition, 95% confidence intervals (95% CI) were calculated for the mean %HR_max_ and mean difference between the levels of each factor (number of players and bouts). Partial eta-squared (η^2^) was used as a measure of effect sizes, and values were interpreted as no effect (η^2^ < 0.04), minimum effect (0.04 < η^2^ < 0.25), moderate effect (0.25 < η^2^ < 0.64), and strong effect (η^2^ > 0.64) [31].

Differences between two vs. two and four vs. four conditions for RPE and TAs were assessed using Wilcoxon signed-rank tests, while differences between bouts for TAs were calculated using Friedman tests. For any significant differences of TAs between bouts, Wilcoxon signed-rank tests were used to perform pairwise comparison [32] and adjusted *p*-values (adj-p) were calculated using a Bonferroni correction. In addition, 95% CIs for the mean differences for the RPE and TAs were calculated via bootstrapping with 10,000 replications. Effect sizes were calculated using the r-value for Wilcoxon signed-rank test [33], and values were interpreted according to Cohen’s benchmarks considering 0.1, 0.3, and 0.5 as a small, medium, and large effect size, respectively [34].

Finally, Spearman’s rank test was used to correlate %HR_max_, RPE, and TAs with GD, PH, and playing experience. Statistical analyses were conducted using the software R (version 3.1.0 for Mac OS X) [35], and the bootstrapping simulations were done using the mosaic package in R (version 0.9.1-3) [36]. The criterion for significance was set at the 0.05 alpha level.

## 3. Results

The mean ± SD (95% CI) of %HR_max_ registered during two vs. two and four vs. four drill conditions were 91.8 ± 3.0 (89.8; 93.7) and 89.7 ± 3.1 (87.8; 91.7), respectively, with a greater value in two vs. two compared to four vs. four (F = 7.758, *p* = 0.018, η^2^ = 0.414 (moderate effect), mean difference (95% CI = 2.059 (0.432; 3.687)).

**Figure 1:**
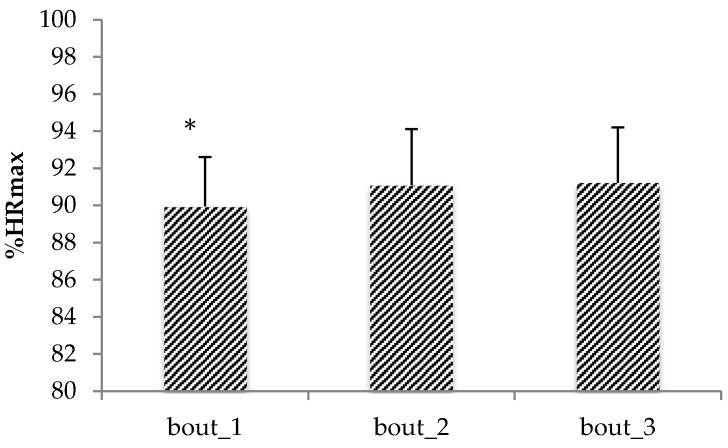
Mean ± SD of %HR_max_ across bouts combining both two vs. two and four vs. four. * indicates a significant difference (adjusted *p* value (adj-p) = 0.043) between first and second bout.

Moreover, results showed a mean and SD (95% CI) %HR_max_ of 89.9 ± 2.7 (88.2; 91.6), 91.1 ± 3.0 (89.2; 93.0), and 91.2 ± 3.0 (89.3; 93.1) for the first, second, and third bout, respectively. A main effect was found for the bout factor (F = 6.720, *p* = 0.005, η^2^ = 0.379 (moderate effect)). Bonferroni post-hoc analysis showed a significant difference between first and second bout (adj-p = 0.043; mean difference (95% CI) = −1.145 (−2.254; −0.035)), a marginal statistical difference value between the first and third bout (adj-p = 0.070; mean difference (95% CI) = −1.282 (−2.658; 0.093)) (Figure 1), and no significant difference between the second and third bout. In addition, no interaction was found between the factors number of players and bouts. Furthermore, a significant difference was found for RPE values (*p* = 0.042; r = 0.408 (medium effect)) between two vs. two (9.3 ± 0.9) and four vs. four condition (8.4 ± 0.9) with a mean difference (95% CI) of 0.9 (0.2; 1.6).

Results related to technical actions are shown in Table 1. A greater number of dribbles (*p* = 0.007; r = 0.560 (large effect)), shots (*p* = 0.003; r = 0.624 (large effect)), and rebounds (*p* = 0.006; r = 0.572 (large effect)) were observed in two vs. two compared to four vs. four condition (Table 1). Moreover, significant differences in pass actions were observed between bouts (*p* = 0.020). Post-hoc analyses highlighted a higher number of passes in the first bout compared to second (adj-p = 0.036; r = 0.512 (large effect)) and third bouts (adj-p = 0.045; r = 0.499 (medium effect)), while no difference was reported between second and third bouts (adj-p = 0.909).

Finally, no significant correlations were found between players’ GD, PH, and playing experience with their %HR_max_, RPE, and TAs (Table 2).

## 4. Discussion

The main findings of this study showed that: (1) the number of players involved influenced the physiological and technical loads during BDs in young (i.e., <15 years of age) basketball players; (2) changes across consecutive bouts of the same drill were observed for the physiological demand, while no changes were found for most of the technical actions; (3) players’ self-assessed maturation status did not correlate with players’ physiological, perceptual, and technical demands in each considered BD, rejecting our hypothesis.

Several variables, such as the use of different rules, court size, and number of players involved can influence the physiological demand of basketball BDs [11,12,13,14]. Previous studies investigating BDs in young elite and sub-elite players in their late adolescent phase reported a moderately higher physiological and perceptual demands in two vs. two (%HR_max_: 86%–87%; RPE: 8–9 AU) compared to four vs. four (%HR_max_: 83%–84%; RPE: 6–8 AU) [13,14]. This is the first study utilizing a multifactorial approach to identify the variables associated with the basketball BDs workload in considering players’ maturity stages. The analysis of the factor number of players involved indicated results in line with those reported in previous investigations showing a moderately higher %HR_max_ and RPE in two vs. two compared to four vs. four condition. The increased physiological and perceptual demands are likely the result of an increased amount of work performed, due to an increase in the relative playing area per player [13,14]. Interestingly, the study of Conte et al. [13]—in which the same ball-drill setting was adopted (three bouts of 4 min interspersed by 2 min of passive recovery)—documented a similar %HR_max_ (two vs. two: 92% vs. 90%; four vs. four: 90% vs. 87%) and an equal RPE (two vs. two: 9 AU; four vs. four: 8 AU) in both conditions. Overall, these results indicate a consistent psychophysiological response during basketball BDs played with a different number of players.

While sport research has deeply focused on the physiological and perceptual demands in basketball BDs, less data are available on the effect of these training methodologies on the technical demand. Previous investigations analyzing BDs technical demand in young elite [14] and non-elite [13] basketball players reported a moderate to strong higher number of technical actions (dribble, rebound ball screen, and close-, mid-range, and 3-point shots) in two vs. two compared to four vs. four condition. Likewise, in the current study, a higher number of dribbles, shots, and rebounds with a large effect size in two vs. two compared to four vs. four in young basketball players has been shown. This result could be explained by several factors. Less skilled (i.e., non-elite) players or less experienced players are likely to have a lower shooting percentage, resulting in more rebounds and second shots. In addition, fewer players for passing options would likely result in more dribbling to move the ball up the court. Finally, greater dribble penetration to score in one vs. one situations is more likely to occur in the two vs. two condition compared to the four vs. four one.

One of our results did not agree with that shown in previous investigations [13,14], as no statistical differences were found when considering the number of passes between two vs. two and four vs. four conditions. This could be explained by the fact that although players performed more ball touches and had more possibility to pass the ball in the two vs. two condition, there were more likely passing solutions in the four vs. four condition. In fact, a higher number of teammates would likely create more possibility to effectively pass the ball. Furthermore, in the two vs. two condition, players likely preferred to play more individually in a one vs. one configuration due to a greater maneuvering area rather than passing the ball compared to the four vs. four condition. This result underlines that basketball coaches should probably use different variables in BDs in order to better train players’ pass skills. Overall, it could be speculated that the variable number of players is effective in modifying the BDs training intensity for young players with different maturation levels.

A novel aspect of this study was the analysis of the variation of physiological and technical responses across bouts. Results showed a statistically lower %HR_max_ in the first bout compared to the following ones. Specifically, the results showed a significant difference between first and second bouts and a marginally significant difference between first and third bouts. This result is similar to that reported in soccer SSGs [16,17,18], and could be explained by the fact that the initial bout is associated with a gradual increase in heart rate to levels required for an aerobic training stimulus. In the second bout, the HR is likely to increase more rapidly because of the stimulus from the previous bout of exercise and the short recovery period. The short recovery periods would limit the return of HR to baseline levels, leading to elevation of HR to the desired level [37]. These results are in contrast with those reported by Castagna et al. [11], in which no statistical differences were found between bouts. The reason for this result may be the shorter recovery period used (2 min) in our study compared to that reported in the previous investigation (3 min). This contrasting result indicates the necessity of future research exploring the effect of different recovery periods between bouts on the physiological demand in basketball BDs.

The analysis of technical actions documented no differences throughout bouts in overall TAs, a result in agreement with previous investigations in soccer [2,16]. In the current study, the pass action was the only TA that reported a decrement across bouts. Likely, this result may be related to the playing style adopted, and perhaps the trend of increased dribbling that was observed from bouts two to three. In fact, no particular indications were provided about playing style, but rules restricted players to use man-marking defense and not zone defense. Therefore, players could decide to use both passing and dribbling actions in order to advance the ball on the court.

This is also the first study investigating the correlation between self-assessed maturation status and physiological and technical demands of basketball BDs. While maturation status influenced young basketball players’ strength outputs and individual time-based technical skills tests (dribbling and defensive movements) [23], no correlation was found in our work between self-assessed maturation status and physiological aspects and technical actions assessed in a game-based condition. This data is in line with those reported in soccer small-sided games [16] in which no correlations were found between players’ maturation status and both exercise intensities and technical scores. Therefore, it can be concluded that BDs elicit a similar training stimulus regardless of players’ maturation status in this age group (under 15). This information seems to be crucial for basketball coaches, considering that youth teams are usually composed by players with the same chronological age but different maturation levels.

In addition, no correlation was reported between the player’s playing experience and the physiological and technical demands in basketball BDs. It was expected that players with greater exposure to competitive basketball would show higher involvement during BDs showing greater physiological and technical demands. Interestingly, in our study, players with more years of training experience were involved to the same degree as novice athletes. This aspect could be explained by the fact that the BDs present a wide range of playing situations that could help players with fewer years of training to enhance their playing experience. This result could suggest to basketball coaches to use this training methodology independently from players’ training background.

One of the limitations of this study is that only 12 players were analyzed and only a self-assessed Tanner stage questionnaire was used to determine players’ maturation status; thus, future research should use a larger sample and several more objective ways to determine players’ biological age. In addition, this study analyzed a specific sample composed by 14-year-old players, while future investigations should be provided on players with a different chronological age (i.e., 12–13 years). Moreover, considering the contrasting results between this study and previous investigations on passing skills in basketball BDs, future investigations are warranted in this area. Finally, future studies should also correlate players’ skill ability levels with BDs physiological and technical demands.

The high HR observed in this study suggests that basketball coaches can use BDs to train physical fitness simultaneously with the technical and tactical skills. In particular, coaches should use the two vs. two condition to impose greater internal load on their players when the periodization of training plans allows it. Furthermore, training sessions including BDs should be used to develop young players’ individual skills in a game-based condition. Specifically, the two vs. two should be preferred to the four vs. four in order to train dribble, shot, and rebound actions. Conversely, passing skills and the ability to intercept or steal the ball or to induce a turnover were not influenced by the different number of players involved, suggesting that coaches should use a different variable to modulate the learning of these abilities. Although the two vs. two condition elicited a higher number of technical actions, basketball coaches should also consider using four vs. four, because it likely stimulates better decision-making ability skills in young players due to more complex interactions with a greater number of players and opponents [13,14]. Considering the differences in the physiological demands across bouts, coaches should carefully plan their practice using specific exercise and recovery periods within a training session that could produce an effective stimulus for aerobic training. In particular, the shorter recovery bouts will likely result in higher workloads and heart rates, inducing a stronger physiological stimulus. Finally, basketball coaches should consider game-based conditioning drills a useful methodology to train the physiological and technical demands of young players with different maturation statuses and playing experiences.

## 5. Conclusions

This study improves the knowledge about the use of BDs in young basketball players, suggesting that BDs are a viable training method that can be used by basketball coaches to train both players’ fitness and technical skills. The different number of players involved affected the physiological and technical demands of BDs in basketball, with a higher training load corresponding to a reduction of the number of players involved. Moreover, physiological demands change across bouts, while no variations were observed for the overall technical actions. Lastly, players’ maturation status and playing experience did not correlate with BDs physiological and technical demands in young basketball players.

## Figures and Tables

**Table 1:** Mean (SD) and *p*-value of technical actions performed in two vs. two and four vs. four conditions and across bouts. Mean difference (95% confidence interval) between two vs. two and four vs. four condition is also reported.

Technical Actions	Number of Players					Bouts			
	Two vs. Two	Four vs. Four	*p*	Mean Difference (95% CI)	r-Value	Bout 1	Bout 2	Bout 3	*p*
Dribble	22.2 (5.8)	13.5 (8.2)	0.007	8.7 (4.5, 12.8)	0.560 (large)	11.7 (4.6)	11.5 (4.4)	12.5 (4.1)	0.218
Pass	21.4 (5.0)	19.2 (6.6)	0.195	2.3 (−1.5, 6.0)	0.272 (small)	15.0 (3.5)	13.2 (2.9)	12.3 (3.9)	0.02
Shot	16.7 (7.2)	5.6 (2.3)	0.003	11.1 (7.3, 14.9)	0.624 (large)	6.7 (2.5)	8.2 (3.5)	7.2 (3.1)	0.142
Interception	1.7 (1.1)	1.4 (1.6)	0.683	0.3 (−0.8, 1.3)	0.091 (no effect)	1.4 (1.4)	0.9 (1.1)	0.7 (0.9)	0.497
Steal	0.9 (0.9)	0.9 (1.0)	0.901	0.0 (−0.7, 0.7)	0.036 (no effect)	0.7 (0.8)	0.7 (0.9)	0.4 (0.7)	0.572
Rebound	7.4 (3.4)	3.1 (1.9)	0.006	4.3 (2.5, 6.2)	0.572 (large)	3.3 (1.8)	3.8 (2.1)	3.3 (2.1)	0.629
Turnover	2.3 (1.7)	2.1 (2.0)	0.781	0.3 (−1.1, 1.7)	0.064 (no effect)	1.6 (1.8)	1.4 (0.9)	1.4 (0.9)	0.832

**Table 2:** Correlation coefficient (*p* value) of percentage of maximal heart rate (%HR_max_), ratings of perceived exertion (RPE), and technical actions with genitalia development (GD), pubic hair growth (PH), and training age in each ball-drill condition.

Physiological/Technical Demands	Two vs. Two			Four vs. Four		
	GD	PH	Training Age	GD	PH	Training Age
%HR_max_	0.168 (0.602)	0.004 (0.991)	−0.230 (0.472)	−0.080 (0.804)	0.131 (0.686)	0.068 (0.833)
RPE	0.082 (0.801)	−0.155 (0.630)	0.000 (1.000)	−0.610 (0.851)	−0.072 (0.824)	0.092 (0.776)
Dribble	0.092 (0.776)	0.213 (0.506)	0.180 (0.575)	−0.057 (0.859)	−0.017 (0.959)	0.209 (0.514)
Pass	0.460 (0.132)	0.331 (0.293)	−0.421 (0.173)	0.493 (0.103)	0.487 (0.108)	−0.394 (0.205)
Shot	−0.023 (0.944)	0.151 (0.639)	0.284 (0.371)	0.156 (0.629)	0.068 (0.833)	−0.011 (0.973)
Interception	0.142 (0.659)	0.324 (0.304)	−0.050 (0.877)	−0.171 (0.595)	−0.085 (0.792)	0.167 (0.605)
Steal	−0.204 (0.525)	−0.002 (0.995)	0.362 (0.248)	0.000 (1.000)	0.148 (0.646)	0.040 (0.902)
Rebound	−0.015 (0.962)	0.198 (0.563)	0.233 (0.466)	−0.105 (0.744)	−0.181 (0.573)	0.178 (0.579)
Turnover	0.043 (0.894)	−0.186 (0.564)	0.212 (0.508)	−0.160 (0.619)	−0.090 (0.782)	0.189 (0.556)
